# Assisting Multitargeted Ligand Affinity Prediction of Receptor Tyrosine Kinases Associated Nonsmall Cell Lung Cancer Treatment with Multitasking Principal Neighborhood Aggregation

**DOI:** 10.3390/molecules27041226

**Published:** 2022-02-11

**Authors:** Fahsai Nakarin, Kajjana Boonpalit, Jiramet Kinchagawat, Patcharapol Wachiraphan, Thanyada Rungrotmongkol, Sarana Nutanong

**Affiliations:** 1 School of Information Science and Technology, Vidyasirimedhi Institute of Science and Technology (VISTEC), Rayong 21210, Thailand; Kajjana.b_s21@vistec.ac.th (K.B.); jiramet.kin@gmail.com (J.K.); patcharapolw_pro@vistec.ac.th (P.W.); snutanon@vistec.ac.th (S.N.); 2 Center of Excellence in Biocatalyst and Sustainable Biotechnology, Faculty of Science, Chulalongkorn University, Bangkok 10330, Thailand; thanyada.r@chula.ac.th; 3 Program in Bioinformatics and Computational Biology, Graduate School, Chulalongkorn University, Bangkok 10330, Thailand

**Keywords:** quantitative structure–activity relationship (QSAR), machine learning, multitasking, affinity prediction, drug discovery

## Abstract

A multitargeted therapeutic approach with hybrid drugs is a promising strategy to enhance anticancer efficiency and overcome drug resistance in nonsmall cell lung cancer (NSCLC) treatment. Estimating affinities of small molecules against targets of interest typically proceeds as a preliminary action for recent drug discovery in the pharmaceutical industry. In this investigation, we employed machine learning models to provide a computationally affordable means for computer-aided screening to accelerate the discovery of potential drug compounds. In particular, we introduced a quantitative structure–activity-relationship (QSAR)-based multitask learning model to facilitate an in silico screening system of multitargeted drug development. Our method combines a recently developed graph-based neural network architecture, principal neighborhood aggregation (PNA), with a descriptor-based deep neural network supporting synergistic utilization of molecular graph and fingerprint features. The model was generated by more than ten-thousands affinity-reported ligands of seven crucial receptor tyrosine kinases in NSCLC from two public data sources. As a result, our multitask model demonstrated better performance than all other benchmark models, as well as achieving satisfying predictive ability regarding applicable QSAR criteria for most tasks within the model’s applicability. Since our model could potentially be a screening tool for practical use, we have provided a model implementation platform with a tutorial that is freely accessible hence, advising the first move in a long journey of cancer drug development.

## 1. Introduction

Multitargeted therapies by hybrid drugs as multitargeted agents with a concept of “single molecule multiple targets” have been alternatively introduced to overcome the anticancer drug resistance together with improving their effectiveness and safety issues [1,2,3,4]. Properly designed hybrid molecules exhibiting several modes of action promote beneficial approaches for malignancies displaying intrinsic or acquired modification by attacking different hallmarks of cancer [4,5]. In the case of nonsmall cell lung cancer (NSCLC) medication, the US Food and Drug Administration (FDA), thus far, approved some receptor tyrosine kinase inhibitors associated with multitargeted affinity, for instance, ErbB family inhibitor Afatinib, ALK/RET dual-blocker Alectinib, and ALK/MET/ROS1 multi-inhibitor Crizotinib [6,7]. These multitargeted drugs were reported in positive outcomes resulting in more prolonged progression-free survival and reduced lung cancer symptoms [8,9,10,11] even though most of them were not initially designed for the border target interaction [12,13].

Rational drug design for multitargeted strategy has been considered a challenging task for researchers with extensive drug development efforts to achieve the promising agents based on current understanding [5,14]. High-throughput screening of target ligand affinity at the half-maximal inhibitory concentration (IC_50_) via biochemical assay provides information related to cancer cell response [15], which indeed consumes massive resources in large-scale development [16]. Instead, virtual high-throughput screening is preferably used in drug design and discovery due to manageable cost and time [17,18]. Ligand-based computational modeling by applying the quantitative structure–activity relationship (QSAR) concept is one of the most promising techniques frequently used to assist the discovery of hit candidates [19,20,21]. The general idea of QSAR is to estimate how strongly the chemical interaction of a ligand performed relies on its structural relevance to learning information [22]. To enable the discovery of hits interacting with multiple targets in conventional QSAR, models are necessarily performed in sequences to filter chemicals in a library by each desired target criteria leading to a limited number of hits passing through the final filter [23]. Multitasking QSAR employing a machine learning-driven model is a methodology that allows synchronous learning by exploiting extracted patterns from one task to aid learning of related tasks [24]. In other words, the chemical properties of molecules that target one protein can be used to infer the properties against another protein from the same family or with a comparable binding site. Thus, multitask QSAR modeling would be an applicable assistant tool [25] to accelerate the discovery of lead candidates targeting multiple tyrosine kinases for NSCLC treatment.

The conventional machine learning models, including linear models such as support vector machine (SVM) and nonlinear models such as random forest (RF) and gradient boosted regression trees (GBRT), are general methods for QSAR study [26]. The molecular structures are transformed into binary vectors, known as molecular fingerprints, which are then employed as model inputs [27,28]. SVM creates the maximum-margin hyperplane in multidimensional feature space, while RF and GBRT build the decision trees, for regression or classification. The main difference between RF and GBRT is that RF applies bagging on multiple decision trees and averages the output from those trees [26], in contrast, GBRT creates a single decision tree sequentially based on the previous tree’s error [29]. However, the learning algorithm modification is required to build a multitasking QSAR model, so the deep neural networks (DNN) framework offers a more straightforward approach to multitask learning on molecular fingerprints. Another powerful method to represent molecular structure in deep learning models is graph representation, which aims to preserve the natural representation of the chemical structure. The atoms and bond connectivity encoded by the atom and bond feature vector are used as input to graph neural networks (GNN), i.e., graph convolutional neural networks (GCN) and graph attention networks (GAT). The GNN’s characteristic is its ability to automatically learn task-specific representations without the use of handcrafted fingerprints [26]. Nevertheless, to determine the sort of model to be used, task-by-task evaluation is required [30].

The GNN has been active in the research development that persuaded beneficial movements in various fields of study, especially molecular machine learning [31,32,33]. However, the current GNN is limited due to isomorphism tasks. The learning power of GNN is restricted by a single aggregator, which is incapable to extract enough information from the neighborhood nodes to differentiate the topologically identical molecules. For instance, the nonidentical molecules, decalin and 1,1-bicyclopentane (Figure S1 in the Supplementary Materials), can be recognized as the same molecule in molecular graph network due to their identical topology [34]. This issue resulted in a lower predictive power of the model [35], causing a difficulty with bioactivity prediction [36]. Accordingly, multiple aggregation strategies have been proposed to improve the GNN’s performance, leading to the development of the principal neighborhood aggregation (PNA) method by DeepMind [37]. PNA is a novel GNN architecture with multiple aggregation layers that are able to distinguish the isomorphic graphs. By comparing with other GNN architectures, PNA showed an outstanding performance in computer vision benchmark (CIFAR10 and MNIST), chemistry benchmark (ZINC), and multitask artificial benchmark datasets [37].

Based on recent studies of PNA, there is no published work of PNA-related QSAR models. Herein, we aimed to develop the first PNA-implemented multitask QSAR model for the prediction of ligand affinity values of seven FDA-drug-targeted receptor tyrosine kinases: ALK, EGFR, HER2/ERBB2, HER4/ERBB4, MET, RET, and ROS1. The ligand features presented in structural fingerprints and molecular graphs were extracted from more than 10,000 reported data on protein-based biochemical assays for the model development. Our model was benchmarked and compared against other QSAR existing models, which once were state-of-the-art models for both descriptor-based and graph-based models. In addition to ensuring the prediction reliability in practical hit-screening, we proposed a prompted method to scope the boundary of model applicability, assisting the model users to evaluate their multitargeted candidates for these seven NSCLC-related tyrosine kinases assuredly.

## 2. Results and Discussion

### 2.1. Overview

**Figure 1:**
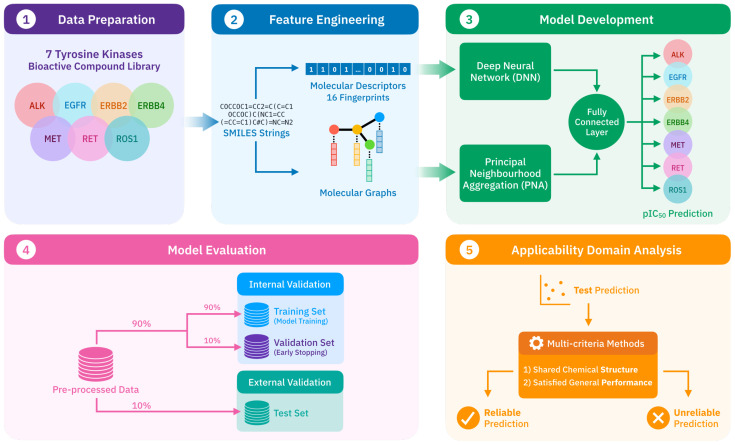
Framework diagram illustrates the workflow process in this study.

An arrangement of a framework developed QSAR model (Figure 1) was designed consisting of the following steps: (1) collecting and preparing affinity profiles of tyrosine kinase inhibitors in pIC_50_, (2) generating descriptor-based and graph-based features that comprehensively represent chemical structures of inhibitor compounds, (3) implemention of our developed multitask deep learning regression model, (4) assessing prediction performance of the model with cross-validation and independent test set, and (5) defining the model applicability for practical implementation.

### 2.2. Data Distribution

A set of 16,345 unique compounds consisting of 11,070 molecules from ChEMBL and 14,130 molecules from BindingDB remained in the curated dataset with 18,561 bioactivity-reported data for the seven studied tyrosine kinases. The pIC_50_ values were not reported for all target proteins in every compound. The EGFR-measured compounds were the most abundant endpoints in the dataset with 7427 points, followed by MET (3618 points), RET (2985 points), ERBB2 (2313 points), ALK (1871 points), ERBB4 (196 points), and ROS1 (151 points) measurement.

**Figure 2:**
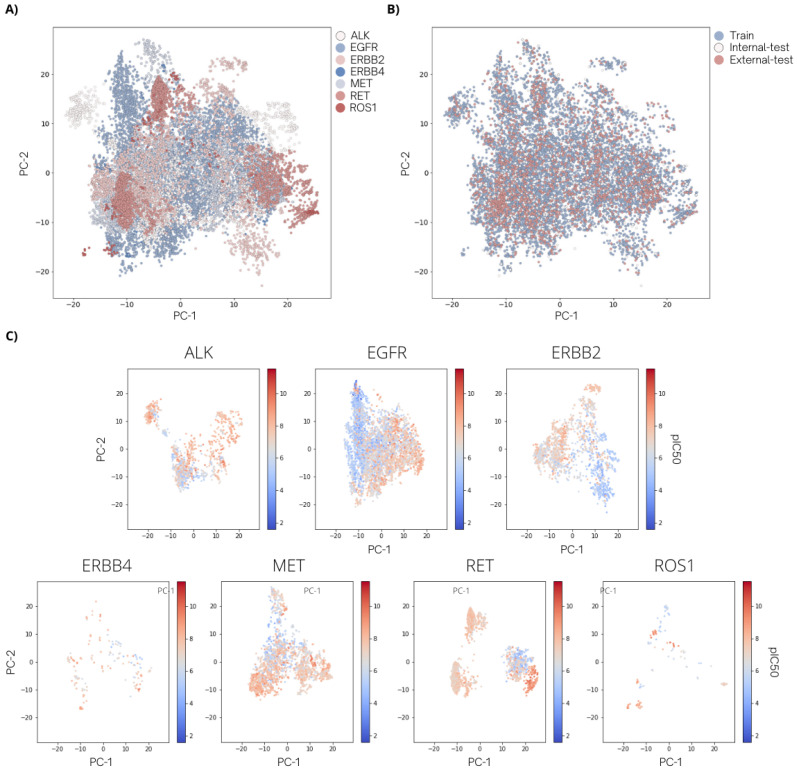
Chemical distributions of the dataset after curation specified by (**A**) target activity and (**B**) divided datasets used in this study. (**C**) Two-dimensional PCA plots for seven tyrosine kinase datasets after AC analysis with pIC_50_ values presenting in color scales; the greater the values, the more potent the compounds to the particular targets.

The meaning of a predictive multitask model based on related knowledge in the tasks is available in the model-building dataset [24]. This assumption requires evidence of activity overlap of all target datasets [38]. Therefore, the two-dimensional visualization of chemical space distribution in each target-reported set was generated by principal component analysis (PCA) from the 16 fingerprints to evaluate the shared structural spatiality across the target domains. The plot in Figure 2A illustrates the overlapping space of all targets between −30 to 30 of PC-1 and PC-2 on the horizontal and vertical axes. The nearby distribution within the certain PC range and 1918 multiple activity-reported compounds (see Table S3 in the Supplementary Materials) indicates a promising movement [39] when combining these seven tyrosine kinase compound sets in QSAR modeling. As shown in Figure 2B, the chemical space of training, validation (internal test), and external test sets display a similar spatial distribution in all target data space, implying a rationally random division method was applied to the dataset.

After identification and eradication of activity cliff (AC) generators by the mean of the activity–similarity difference in the model-building dataset (train–validation set), the chemical space of each target set was visualized by a PCA plot (Figure 2C) with its activity profile. Overall, the compounds in their target space were exhibited a less overlapping chemical space between the red-zone active (pIC_50_ value > 7) and the blue-zone inactive (pIC_50_ value ≤ 7) compounds compared to the data plots before identified AC-pair removal (Figure S2 in the Supplementary Materials). This characteristic should benefit the modelability [40] of QSAR modeling by supporting the critical strategy of QSAR that is based on similar properties probably observed in structurally similar chemicals. Details of the AC analysis method relating to calculating ACs and excluding the identified ACs was described in the Supplementary Materials.

### 2.3. Comparison of Machine Learning Algorithms

**Figure 3:**
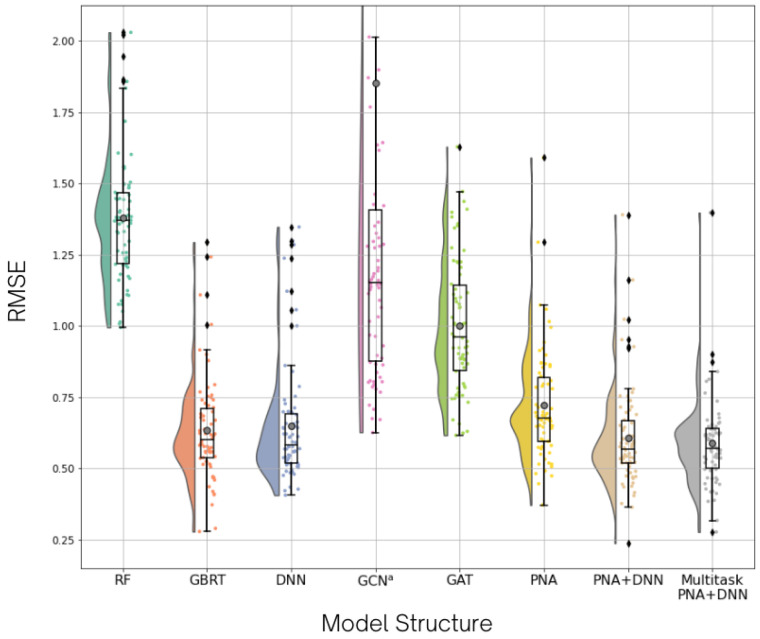
Benchmark model comparison. In this graph, each model structure consists of all RMSE values from 10-fold cross-validation for all targets. The RMSE distribution is shown in a half-violin plot and box plot, which shows quartiles 1, 2, and 3 of the distribution. The mean value of each model is marked as a grey dot inside the box plot. ^a^ Note that the graph does not cover all RMSE points in the GCN plot, this is due to an extensive spread of the outlier data in the RMSE range of 6.5–8.5.

To compare the multitask model performance with baseline algorithms, the average root-mean-square error (RMSE) over 10-fold cross-validation was collected from every benchmark model (Table 1) and the box and violin plots of average RMSE values from seven target predictions for all models are presented in Figure 3.

The GBRT model shows the lowest overall RMSE among the descriptor-based benchmark models (RF, GBRT, and DNN) with the average overall RMSE of 0.6348. However, the RMSE of DNN model is lower than GBRT in five out of seven targets. For the graph-based benchmark models (GCN, GAT, and PNA), PNA is the best model with the all-target RMSE of 0.7211. The RMSEs of the PNA model are lower than those of the other models in all targets. We introduced DNN to the PNA model as it might enhance the descriptive power of molecular representation by integrating graph-based and descriptor-based models and capture the pattern from both molecular graphs and fingerprints [28]. Thus, this method could lead us to better model performance [41,42,43]. For a combined PNA model with DNN, the improvement of the model is indicated. The overall RMSEs decreased from 0.6488 and 0.7211 in DNN and PNA to 0.6072 in PNA+DNN. As the activity overlap was found in data distribution, the multitask learning method was applied on the PNA+DNN model and the results show that the all-target RMSE is reduced to 0.5883. Although the RMSEs of five tasks are slightly increased from the single-task PNA+DNN model, the RMSEs of ERBB4 and ROS1 tasks, which faced data lacking, are decreased by around 0.05 and 0.10, respectively. These results indicate the influence of multitask learning in the data-lacking tasks, while the overall performance of the model is likely maintained.

### 2.4. Model Results and Validation

The multitask model consisting of PNA and DNN algorithms was employed for the prediction of pIC_50_ against seven tyrosine kinases targets. The statistical results of the developed QSAR model in numeric RMSE and *R*-squared formations are shown in Table 2 to evaluate if the model has achieved predictive power conditions. While RMSE is a standard method to compute errors of quantitative prediction from normalized distances between observed values and predicted values, *R*-squared (*R*^2^) indicates how well a model describes the variation of the responses overall. However, it does not successfully present the explanation in a particular dataset. Alternately, RMSE is often used to indicate a model’s usefulness in a regression task [26,30]. An error-based metric as RMSE, on the other hand, indirectly presents predictive achievement of the modeling process, while a direct estimation of a QSAR model is counted by *R*-squared [44]. In this research, the two metrics, *R*^2^ (or *Q*^2^ in cross-validation) and RMSE, were satisfied to determine the model performance in discrete roles.

**Figure 4:**
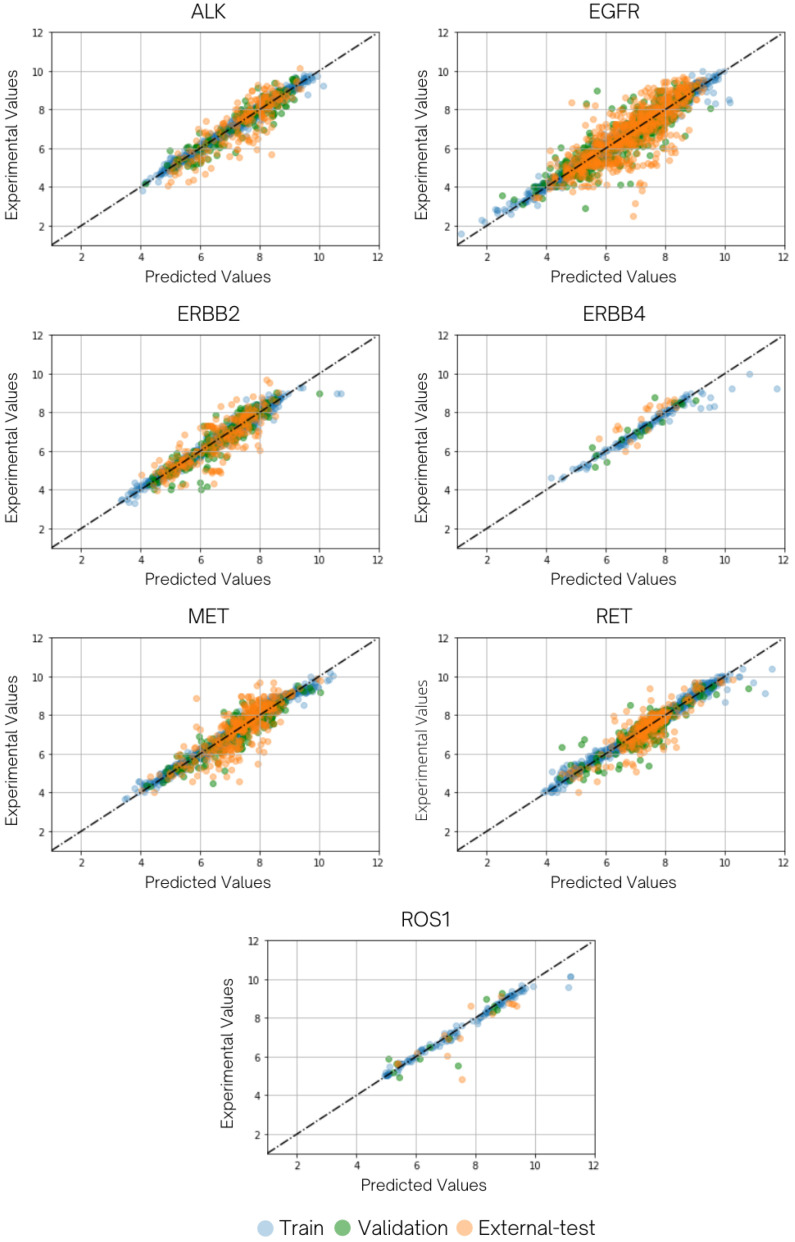
The plots display the distribution of model-predicted values versus experimental values of the seven tyrosine kinases with ideal dashed trendlines (y = x). The training set, validation (internal test) set, and external test set are represented by light blue, green, and amber marks, respectively.

Through tenfold cross-validation, except for ERBB4, all target activity predictions displayed good predictive results with *Q*^2^ around 0.74 to 0.88, relatively higher than a recommendation from QSAR practitioners at 0.7 [45]. Accordingly, low RMSECV (RMSE from cross-validation) results in satisfied-*Q*^2^ tasks presented as 0.463, 0.527, 0.558, 0.563, 0.594, and 0.663 for RET, ROS1, MET, ERBB2, ALK, and EGFR, in order. In contrast, ERBB4 with RMSECV and *Q*^2^ of 0.8167 and 0.4517 was unable to be considered as a satisfying performance. Despite the fact that the evaluation metrics in the training dataset are considerably higher than those for internal validation, this situation would not certainly imply a poor QSAR model in the case of manipulating a deep neural network [46]. The training–validation loss ratio could serve as a heuristic to indicate overfitting in some instances, what constitutes a suitable threshold may differ according to the model type and the dataset. The training–validation loss ratio could serve as a heuristic to indicate overfitting in some instances, what constitutes a suitable threshold may differ according to the model type and the dataset. Various machine-learning models, especially in intricate architectures such as deep learning, have been found to be a practical approach, even when the ratio between training loss and validation loss is high [47,48,49]. A well-established phenomenon in deep learning, as well as some classical machine learning, has addressed this issue regarding the bias-variance tradeoff, known for the double descent risk curve [50]. Specifically, the test and validation performance increase upon adding parameters, even if it would mean that the training loss could go to a very low range. To ensure this, we compared our model performance with the same architectural models, yet different in the degree of regularization. The results emphasize that our current model provides the best bias–variance tradeoff among other hyperparameter configurations (Figure S4 in the Supplementary Materials). In addition, the consideration of overfitting in deep learning is generally counted when models are either overly parameterized or overly trained to the point of the validation loss starting to increase. By the meaning of early-stopping practice, our model did not experience an increasing trend in the validation loss, suggesting that the model should not be considered facing a concern in overfitting issue (Figure S4A in the Supplementary Materials). The external validation performance on the independent data supports the insistence that the model captured the general relationship of the several targets used in this study. Overall, the *R*^2^ values for all targets, except ERBB4, were reported between 0.62 and 0.73, which are greater than the standard *R*^2^ threshold for QSAR models of 0.6. Furthermore, other statistical parameters as $\frac{\left(R^{2}-R_{0}^{2}\right)}{R^{2}}$, $|R_{0}^{2}-R_{0}^{{}^{\prime }2}|$, and *k* values of all seven-target tasks (Table 2) are acceptable for certain conditions in the particular substances (see Section 3.6.2). Figure 4 illustrates the clustering of overall data points near the ideal fitting line for training, validation, and test sets for all targets, demonstrating a high correlation between predicted and observed values.

The prediction of absolute activity in the ERBB4 task could suffer from insufficient data in the data source, especially in multitarget reported chemical profiles [39] (approximately 90%) compared to the better prediction in the ROS1 target that provides 100% of chemicals in the dataset involved in multitarget activity. Nonetheless, it can not deny the assumption that a clustering characteristic in a property range of the ERBB4 test set led to insufficient predictive power on ERBB4’s activity prediction pointed by *R*-squared for external validation. Since the difference between RMSE of prediction (RMSEP) from the ERBB4 task and a 10% of the property range in its training set is nearly equal to this variance in the ALK task which exhibited a pleasant data-fitting performance (*R*^2^ = 0.7280). As shown in Figure 4, test data points of ALK distribute between 5 to 10 while ERBB4 experiences a narrow span of around 6 to 8. According to a calculation of *R*^2^, $\sum {\left(Y-\bar{Y}\right)}^{2}$ would be greater if a larger variation in observed values is obtained, leading to a pointless increase of *R*^2^ even if the prediction residuals are maintained [46]. So far, the prediction task of the ERBB4 target could not be considered as a predictive QSAR for practical screening. However, if we acknowledge that statistical fluctuations easily influence a small dataset during random data partitioning [51] along with RMSEP, the ERBB4-activity prediction from our multitask model tends to produce a promising result when further coupling with a more appropriate property range. Note that a 10% of training range of each task can be found in Table S8 in the Supplementary Materials.

### 2.5. Applicability Domain Analysis

A question arises in the reliability of an individual model result if a specific compound prediction is tested in actual screening practices. Many QSAR models have proven a concept of the applicability domain (AD) and shown significant improvement in the prediction result when including a defined domain of applicability [52,53]. QSAR practitioners have developed multiple techniques to define the applicability of QSAR prediction models [54]. However, AD methodology should cover broader perspectives rather than feature sharing for critical decision making. In this study, we adopted the elaborated AD concept from Hanser, Thierry, et al. [55] to create a condition inside the domain of our model applicability. We also considered significant chemical structure overlapping in test and training chemicals to ensure that the predicted activities are determined by model learning. Meanwhile, general performance within the test compounds’ structural space is taken into account to evaluate the tendency of test results to serve reliable predictions.

**Figure 5:**
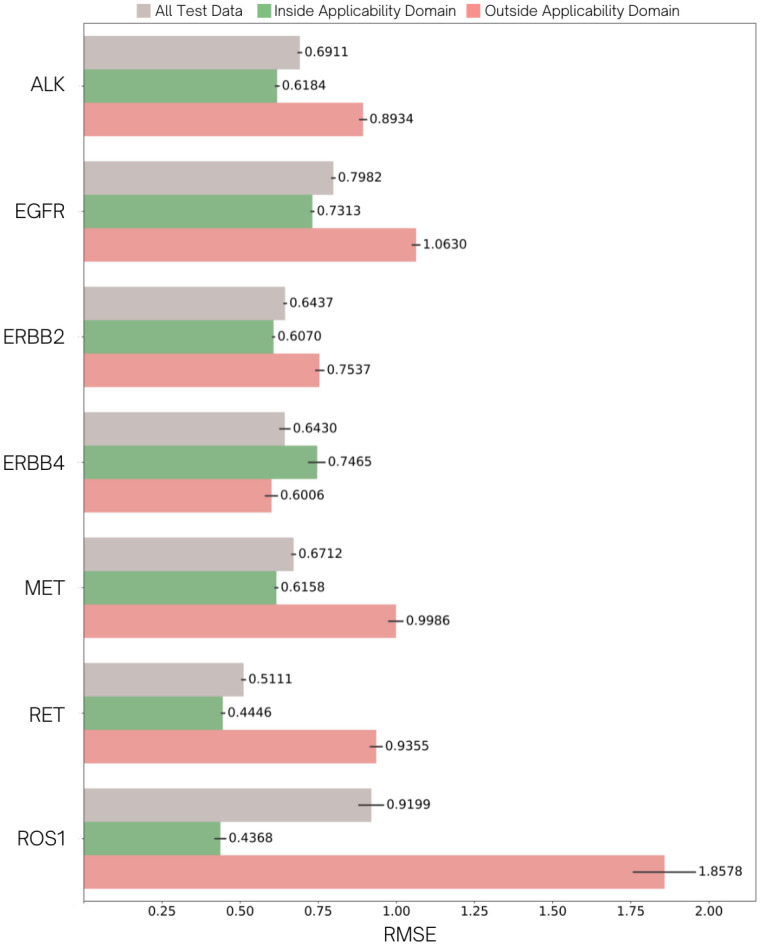
The bar graph presents the influence of the defined applicability domain to external test activity prediction of seven tyrosine kinases in RMSE. Each target consists of three bars, which are denoted for all test datasets (grey), test compounds considered inside (green), and outside (pink) applicability domain. The error bar represents the 95% confidence interval of the variation in predictive performance with 20 different random seeds. The numbers beside each error bar indicate the average RMSE over 20 times of test prediction.

Our defined AD method was examined in external test prediction using the developed multitask PNA+NN model with 20 variations of training random seeds to determine the confidentiality of the prediction scope and to reveal the actual predictive ability of the model. Although predictive power of most prediction tasks fulfilled the QSAR multicriterion without AD implementation, the performance inside target domains was improved in the certain tasks indicated by the decrease of their RMSE values, as shown in Figure 5. On the other hand, the outside-domain compounds encountered an overall poor performance for most tasks even compared to their abilities before implementing the AD conditions. Enhancement of the test’s prediction by AD also corresponded with *R*^2^ that performed in a range around 0.68–0.88 for all satisfied-criteria tasks (Table S6b in the Supplementary Materials). In contrast, the test set considered inside the ERBB4 domain was observed in a more flawed prediction than the outside domain set. Thus, the acceptable model is necessarily proved to secure the benefits of an AD approach in the prediction. Optimized parameters involving the AD method can be found in Tables S7 and S8 in the Supplementary Materials.

### 2.6. Online Screening Service and Model Implementation

ML-driven virtual screening serving as a high-throughput process provides more cost and time manageability in an extensive library contrasted to traditional methods for drug discovery [17,56]. Our developed multitask model had been plausibly established to assist a multitargeted hit-finding for ALK (*R*^2^ = 0.7575), EGFR (*R*^2^ = 0.7082), ERBB2 (*R*^2^ = 0.7090), MET (*R*^2^ = 0.6783), RET (*R*^2^ = 0.7396), and ROS1 (*R*^2^ = 0.8794) with the defined domains of applicability. The designed model coupling with the AD method is available at https://github.com/kajjana/Multibind-RTKs (accessed on 23 December 2021), to serve as an online screening platform for the identification of these crucial tyrosine kinase inhibitors involved in NSCLC therapy [57]. We have also provided a detailed tutorial to guide users step-by-step while utilizing our tool. Users are required to prepare a candidate library with molecular representations in a desalted Simplified Molecular-Input Line-Entry System (SMILES) format. The output containing pIC_50_-activity prediction of seven tyrosine kinases would be generated with an assigned AD presenting either “inside” or “outside” for each target. Examples of input and output files are provided in the same source. All parameters used in the calculations are fixed at the best value from our optimization. Most of the prediction tasks (6 out of 7) certainly afforded pleasant power in numeric prediction; instead, ERBB4 activity prediction has not been recommended to identify and prioritize hit compounds due to its suspected predictive performance. In addition to the prompted screening model, a training version of the multitask modeling is provided for free access at the corresponding link to enhance flexibility in training with applicant’s sources of interest, which promotes the most fitting prediction for special needs.

## 3. Materials and Methods

### 3.1. Dataset

In this study, we collected the experimental IC_50_ values from two publicly accessible databases, ChEMBL and BindingDB, to maximize the variation of dataset in model building and validation process. Our integrated dataset contains bioactivity data of compounds of the wild-type *Homo sapiens* ALK, EGFR, ERBB2, ERBB4, MET, RET, and ROS1, which are the tyrosine kinases reported as targets of FDA-approved drugs for NSCLC treatment [6].

### 3.2. Data Curation

The initial dataset was curated following a protocol from Virakarin, Puri et al. [43]. In brief, compound structures in the dataset were presented in SMILES format. Each compound provides information covering details about its target, assay description and activity value. Only compounds reported with continuous IC_50_ values from biochemical or single protein assays with the interest target were collected. The redundant compounds with the same assay description and IC_50_ were removed. The lowest IC_50_ value was retained as an endpoint if the compound reported different activity values. Salts were stripped from SMILES format for all compounds. To avoid confusion in different units, we converted all IC_50_ values in the molar unit to -log(IC_50_) referred to as pIC_50_. An additional outlier removal proceeded in ChEMBL data using the interquartile range (IQR) cutoff method of the ChEMBL-provided parameters, considering Lipinski’s rule of five violations, molecular weight, and octanol–water partition coefficient (logP). The compound qualified was considered as an outlier if it fell outside the range of Q_1_ − 3(IQR), Q_3_ + 3(IQR), and was excluded from each target dataset. After curation, compound sets from two sources were merged, leaving only one better-reported value (higher pIC_50_) if similar compounds reported redundant activities. The final data resulted in 16,345 compounds with at least one activity value focused against seven tyrosine kinases; then, were brought into further steps. The summarized number of compounds divided by reported activities for each target from two data sources are shown in Table S1a in the Supplementary Materials.

### 3.3. Data Split

To create a dataset for training and testing the model with a multitasking structure, we randomly divided the data by remaining the ratio in the number of candidate data points per task to the original dataset. In order to define a target label of each compound with more than one activity value, the targets of that compound were ranked in ascending order by the number of reported compounds in each target data. The labels of the multitarget compounds were dedicated to the target with more minor reported data. An external test set, which plays as an independent test set (unseen data), was taken out for 10% of the dataset at the beginning. The remaining 90% of the training–validation set was further divided into a training set and a validation set in 90:10 ratio. The validation set, or internal test set, was made to terminate deep neural network models in an early stopping process. The datasets of single-task models were further separated into each target domain from the same three subdatasets of the multitask model data. The number of each subdataset for multitask and single-task modeling is listed in Table S1a in the Supplementary Materials. Then, we removed AC generators from the training–validation set of each target to smoothen the activity landscape for building a model with less confusing data [58].

### 3.4. Feature Extraction

In this work, we represent chemical structure data as descriptors and graphs. The use of feature types depends on the input characteristics of model algorithms. For molecular fingerprints, we generated 16 structural fingerprints from SMILES through freely available platforms from the CDK [59], RDKit [60], and PyBEL [61] packages and stored them in Boolean data type. Details of the selected fingerprints are provided in Table S2 in the Supplementary Materials. Feature reduction with PCA method reduced the 16-descriptor dimensions by remaining 95% of the initial variance in training data. For a graph featurization, each canonical SMILES was constructed into a binary vector of 30-dimension node (atom) and 75-dimension edge (bond) by RDKit [60] implemented in DeepChem [62]. The feature length was set by following a default of DeepChem [28].

### 3.5. Model Construction

The multitask model was constructed using PNA layers to empower GNN, learning on molecular graphs. Multiple aggregators were employed to extract adequate information from the neighborhood nodes and to ensure that at least one aggregator was compatible with our task [63]. The DNN layers were built in parallel to the PNA layers to extract additional features from molecular fingerprints, then integrated with the PNA component via a fully connected layer (FLCs). The model was implemented using PyTorch Geometric [64]. The summary of model architecture is illustrated in Figure 1.

The PNA layer consists of four compositions of the multiple aggregators (mean, standard deviation, maximum, and minimum), whereas most GNN layers simply adopt only the primitive summation aggregator that may cause exploding or vanishing gradients. The three degree-scalers (identical, amplification, and attenuation) are combined with the aggregators to improve the model’s generalization.

(1) $$S\left(d,\alpha \right)={\left(\frac{log(d+1)}{\delta }\right)}^{\alpha },\delta =\frac{1}{\left|train\right|}\sum_{i\in train}log\left(d_{i}+1\right)$$

where *S* is degree-scaler with linear-degree (*d* > 0) and variable parameter (α), which is zero for identical, +1 for amplification, and −1 for attenuation.

The PNA learns the graph representations by accumulating the information of feature vectors of neighborhood nodes. The feature vector $x_{i}^{t},\;x_{j}^{t}$ encodes the atomic properties of nodes *i* and *j* at layer *t*, $E_{j\to i}$ encodes the bond properties of edge (*j*,*i*), and *MLP* denotes multilayer perceptrons (MLPs)

(2) $$x_{i}^{t+1}=MLP\left(x_{i}^{t},\underset{\begin{array}{c} (j,i)\in E \end{array}}{⨁}MLP\left(x_{i}^{t},E_{j\to i},x_{j}^{t}\right)\right)$$

where ⊗ is a tensor product and

(3) $$\begin{array}{c} ⨁=\underset{\mathrm{scalers}}{\underset{︸}{\left[\begin{array}{c} 1\\ S(D,\alpha =1)\\ S(D,\alpha =-1) \end{array}\right]}}⊗\underset{\mathrm{aggregators}}{\underset{︸}{\left[\begin{array}{c} \mu \\ \sigma \\ \max\\ \min \end{array}\right]}} \end{array}$$

The PNA and DNN layers were built independently and applied on molecular graphs and molecular fingerprints, respectively. The pooling layer coupled with MLPs was made on top of the PNA layer, then concatenated with DNN layers. The model resulted in pIC_50_ of the molecule against seven target tyrosine kinases via FLCs. The Adam optimizer with L2 regularization was used to minimize the loss function, and training was stopped early after the internal test (validation) set error and the average of the previous ten epochs’ internal test set error did not decrease after ten epochs.

The multitask learning method utilized for model construction is the hard parameter sharing scheme [65], in which all layers except the last FLCs are shared between each task. Each of the chosen tasks for the model is highly related due to the target belonging in the same group of protein. The model is built in comparison to the same architecture for single task evaluation to demonstrate the effect of multitask learning. The equation for the backward loss is simply

(4) $$Loss=\sum_{t=1}^{T}\left(\frac{1}{n_{t}}\right)\sum_{i=1}^{n}{\left(\hat{Y_{i}}-Y_{i}\right)}^{2}$$

where *T* represent the number of task and $n_{t}$ is the number of the sample of the respective task within the mini batch. $\hat{Y_{i}}$ is the predicted value of the compound *i* and $Y_{i}$ is the experimental value for the particular compound

The model was trained on the joint data of seven tasks, and the missing label of some tasks was allowed. The training was performed in a mixed batch of random order, such that each batch may contain unequal numbers of the sample from each task. The loss utilized for the backpropagation algorithm of the model is the total mean-squared error (MSE) of the existing label within the batch.

To obtain the best hyperparameter set of the multitask model, the Bayesian optimization from the optuna [66] library was performed for the following parameters: (i) number of PNA layer, (ii) number of nodes in MLP, (iii) number of nodes in FLCs, (iv) number of DNN layer, (v) the number of nodes in DNN layer, (vi) probability of dropout, (vii) learning rate, (viii) weight decay rate, and (ix) batch size. Table S4 and Table S5 in the Supplementary Materials provides details of the hyperparameter ranges and best hyperparameter set, respectively.

### 3.6. Model Assessment and Statistical Performance

To assess the performance of the regression models in this study, we performed internal validation by stratified 10-fold cross-validation and external validation with an independent test set. Two statistical parameters were used to evaluate each model’s performance as follows: (i) the standard deviation of the residuals or *RMSE* (Equation (5)), and (ii) the coefficient of determination or *R*^2^ (Equation (6))

(5) $$RMSE=\frac{1}{n}\sqrt{\sum_{i=1}^{n}{\left(\hat{Y_{i}}-Y_{i}\right)}^{2}}$$

(6) $$R^{2}=1-\frac{\sum_{i=1}^{n}{\left(\hat{Y_{i}}-Y_{i}\right)}^{2}}{\sum_{i=1}^{n}{\left(Y_{i}-\bar{Y}\right)}^{2}}$$

where *n* is total the number of chemicals and $\bar{Y}$ is the mean value of all compound samples. The smaller the value of *RMSE* and the closer to 1.0 of *R*^2^, the better accuracy of the model prediction is performed.

#### 3.6.1. Internal Validation

The predictive ability of a fitting model in terms of stability and robustness can be verified by internal cross-validation on the training data [45,67]. The dataset was randomly subdivided into 10 equal-sized subsamples containing similar target-label proportions to original training data as mentioned in the data split step. Nine of the subparts were used for training sets to calibrate the model, and one group was held as a test set. The procedure was repeated in 10 iterations on the remaining 9 training data to evaluate with different omitted training data. The statistical parameters from RMSE (Equation (5)) and *R*^2^ (Equation (6)) were calculated as overall result validation over the 10-round process referred to as RMSECV and Q^2^ in order. The lower RMSECV and the higher Q^2^ indicate that the models have achieved a better consistent and robust prediction capacity [46].

#### 3.6.2. External Validation

The use of an independent test set for external validation is necessary for assessing the reliability of QSAR models outside the training set, hence, demonstrating a practical scenario [44]. Since the test set is not involved in the model generating process, test compounds are unknown to the models. For external evaluation, RMSEP and *R*^2^ are used to assess the model prediction similarly to internal validation.

In addition, the combination of two validation multicriterions recommended by Golbraikh, Alexander, and Tropsha were applied to the model to evaluate whatever our developed QSAR model demonstrates, which showed adequate predictive performance for practical use. According to the latest version of a Golbraikh-Tropsha rule [68,69] and Alexander et al. [44], the acceptable QSAR model must satisfy the following conditions:

1. $Q^{2}>0.5$
2. $R^{2}>0.6$; however, QSAR models can be considered practically applicable if the models exhibiting a low RMSE with independent data
3. $\frac{\left(R^{2}-R_{0}^{2}\right)}{R^{2}}<0.1$
4. 0.9≤k≥1.1
5. $|R_{0}^{2}-R_{0}^{{}^{\prime }2}|<0.3$

where $Q^{2}$ and $R^{2}$ are the same coefficients of determination from cross-validation and external test quantities as previously specified; $R_{0}^{2}$ and $R_{0}^{{}^{\prime }2}$ are the correlation coefficients through the origin of predicted (X-axis) versus experimental (Y-axis) values and experimental (X-axis) versus predicted (Y-axis) values, respectively; and *k* defines as a slope of the test predicted (X-axis) versus experimental (Y-axis) trendline through the origin.

### 3.7. Model Benchmarking

We compared the performance of the multitask model with the selected machine learning methods, which have been reported as a benchmark for molecular machine learning [30] or reported in benchmarking of PNA model [37]. To unveil the performance of PNA, the benchmark models were constructed as a single-task model. The ensemble learning is the traditional ML algorithms with several reported studies on molecular property prediction [30,70,71,72]. RF [73] and GBRT [29] models were constructed using implementation in scikit-learn [74] to apply on molecular fingerprints. Recently, the graph-based models have been gaining attraction as a state-of-the-art method for molecular property prediction since atom-level or bond-level features were used as inputs for the molecular graph. A number of graph-based QSAR models are increased, including GCN [26,30,75] and GAT [26,76,77]. The GCN and GAT models from DeepChem were built to apply on molecular graphs. Additionally, the single-task models of DNN, PNA, and PNA combined with DNN also counted as benchmark models to see the improvement of model via combining PNA with DNN and multitask learning method. The benchmark models were trained using the same method as the multitask model and their hyperparameters were optimized using bayesian optimization from skopt [78] for RF and GBRT models, DeepChem for GCN and GAT models, and Optuna for all other models. Table S4 and Table S5 in the Supplementary Materials provide the details of hyperparameter ranges and best hyperparameter sets of all benchmark models. We also benchmarked our proposed multitask model with all mentioned models by implementing 10-fold cross-validation. An average RMSE value calculated from each approach in the cross-validation was utilized to indicate the model’s predictive ability.

### 3.8. Calculation of Applicability Domain

The Organization of Economic Co-operation and Development (OECD) [67] has introduced the AD to a QSAR methodology to define model boundaries, which are applicable in providing accurate predictions with confidence for query compounds. To apply the AD concept, we have verified that any test molecules could be predicted by the scope of the model’s specification. Dealing with more than ten thousand data points, we intended to choose a similarity-based method [54], which conveniently applies to an extensive dataset for structural comparison. The method quantifies the Tanimoto index through Extended Connectivity Fingerprint with radius 4 (ECFP4) of test compounds to the nearest neighbor in all-target model training compounds. To determine an appropriate threshold of similarity, we optimized the value through the prediction result from cross-validation. We selected the compounds within the third quartile (Q3) range of the squared error distribution in each approach and computed Z-scores of their similarities in the remaining molecules. A threshold value in each fold was determined by the minimum Tanimoto similarity among the compounds holding their Z-scores within a critical Z-value at 95% confidence level. The average of the 10-fold thresholds was further calculated to be an optimized threshold for the applicable model boundary. Suppose the maximum Tanimoto similarity of a test compound to its nearest neighbor has reached the threshold. In that case, the compound is reflected in a meaningful chemical sharing to the model building data. Another perspective of our applied AD condition is to assure that a reliable prediction is exclusively served to model users. The reliability of an individual response was filtered by the relevant information of the model for a specific task. The quality of training knowledge available to the nearby test molecules in the region can be indicated by the general performance of training neighbors when performing cross-validation [55]. The target neighbors surrounding the test chemicals were identified by Tanimoto similarity reaching 0.35 to training chemicals with specific target-activity labels. A mean squared residual of these neighbors was calculated and indicated a general performance around test compounds. The cutoff value identifying a confidential area for each task was settled at 10% of a training property range [46] for a particular target. The test compounds potentially provide a reliable prediction if the mean-squared-error values fall below the target-cutting number.

## 4. Conclusions

In this study, we proposed a framework of QSAR-model development in a deep-learning based multitasking approach from two public data sources with a combination of 16 standard molecular fingerprints and PNA, which is the newly improved molecular graph algorithm, to assist the preliminary step of multitargeted drug discovery to enhance NSCLC targeted therapy. Overall, our multitask model outperformed all baseline models previously used in molecular modeling for both descriptor-based and graph-based architectures, as well as the similar PNA+DNN model structure in single-task. The developed model fulfilled all widely-used predictive QSAR multicriterions through internal and external validations, either with or without the defined AD in 6 out of 7 prediction tasks. The evaluations indicate that our QSAR model provides a satisfying predictive power with the potential of a screening tool for practical usage. In addition, our pre-trained model by learning 7 kinds of protein in the receptor tyrosine kinase group reasonably accommodates useful information to further transfer this knowledge into the related prediction tasks, for example, other tyrosine kinases involved in other cancer treatments.

## Figures and Tables

**Table 1:** Comparison of baseline algorithms by average RMSE over 10-fold cross-validation.

Target Name	Model Structure							
	Single-Task							Multitask
	RF	GBRT	DNN	GCN	GAT	PNA	PNA+DNN	PNA+DNN
All	1.3783	0.6348	0.6488	1.8507	1.0018	0.7211	0.6072	**0.5883**
ALK	1.3912	0.6318	0.6108	1.3069	0.9658	0.6861	**0.5671**	0.5921
EGFR	1.4533	0.7224	**0.6577**	1.2630	1.0958	0.8343	0.6898	0.6612
ERBB2	1.3592	0.5931	0.5603	0.8063	0.8163	0.7139	**0.5567**	0.5592
ERBB4	1.3386	0.8921	1.0511	1.3941	1.1843	0.9004	0.8370	**0.7837**
MET	1.2484	0.5855	**0.5458**	0.9500	0.9244	0.6337	0.5557	0.5579
RET	1.0885	0.4812	0.4778	1.0220	0.6992	0.5522	**0.4486**	0.4582
ROS1	1.7687	0.5373	0.6379	6.2125	1.3267	0.7269	0.5950	**0.5059**

**Table 2:** Statistical results of seven tyrosine kinases activity prediction from our multitask model.

Target Name	Calibration		Internal Validation		External Validation				
	RMSE${}_{\mathrm{train}}$	R${}_{\mathrm{train}}^{2}$	RMSECV	Q${}^{2}$	RMSEP	R${}^{2}$	$\frac{R^{2}-R_{0}^{2}}{R_{0}^{2}}$	*k*	$|R_{0}^{2}-R_{0}^{{}^{\prime }2}|$
ALK	0.1578	0.9840	0.5944	0.7735	0.6771	0.7280	−0.0046	0.9900	0.1489
EGFR	0.1777	0.9831	0.6629	0.7645	0.8083	0.6664	−0.0017	0.9931	0.1288
ERBB2	0.1712	0.9790	0.5627	0.7734	0.6558	0.6973	−0.0011	0.9949	0.0913
ERBB4	0.3266	0.9123	0.8167	0.4517	0.6251	0.4344	−0.3745	1.0457	0.0789
MET	0.1586	0.9795	0.5585	0.7453	0.6723	0.6569	−0.0001	0.9988	0.2038
RET	0.1723	0.9688	0.4629	0.7746	0.5072	0.7192	−0.0019	0.9952	0.0985
ROS1	0.2379	0.9746	0.5274	0.8750	0.8527	0.6219	−0.1047	0.9546	0.0538

## Data Availability

Data and python implementation of the model development are freely available at https://github.com/kajjana/Multibind-RTKs/tree/main/code (accessed on 23 December 2021).

## Supplementary Materials

Supplementary Materials can be found as follows [79,80,81,82,83]. supportingInfo.pdf: Topologically identical structure of decalin and 1,1-bicyclopentane (Figure S1); Two-dimensional PCA plots of train-validation sets for each target before removing activity cliffs with pIC_50_ values (Figure S2); Benchmark model comparison in RMSE values from 10-fold cross-validation (Figure S3); The performance on training and validation data of the model parameters used in this study along with the varied parameters (Figure S4); The influence of the defined applicability domain to external-test activity prediction of 7 tyrosine kinases in R${}^{2}$ (Figure S5); Calculation of Activity Cliffs; Explanation of Online Screening Service (Figure S6–S11); supportingTable.xlsx: Detailed Information of Our Data Set and Its Prediction (Table S1a,b); Molecular Descriptors Used in This Study and Software Implementation (Table S2); Number of Bioactivity Label per Task (Table S3); Parameter Ranges for an Hyperparameter Tuning (Table S4); Optimized Parameter Sets Used for Model Training (Table S5); Applicability Domain Evaluation of External-Test set (Table S6a,b); Applicability Domain Optimization of Similarity Parameter (Table S7); Characteristics of Training Data (Table S8); Details for Target Proteins of Research Interest (Table S9).
