# Supplementary material for: Assisting Multitargeted Ligand Affinity Prediction of Receptor Tyrosine Kinases Associated Nonsmall Cell Lung Cancer Treatment with Multitasking Principal Neighborhood Aggregation
Source: Molecules. 2022 Feb 11;27(4):1226. doi: 10.3390/molecules27041226 (PMC8878292; doi:10.3390/molecules27041226)
Supplement: Supplementary file 1 [file molecules-27-01226-s001.zip › molecules-1518574-supplementary/supplement_checked/supportingInfo.pdf]

Supplementary Materials: Assisting Multi-Targeted Ligand Affinity Prediction of Receptor Tyrosine Kinases Associated Non-Small Cell Lung Cancer Treatment with Multitasking Principal Neighbourhood Aggregation

Fahsai Nakarin*, Kajjana Boonpalit, Jiramet Kinchagawat , Patcharapol Wachiraphan, Thanyada Rungrotmongkol and Sarana Nutanong

*

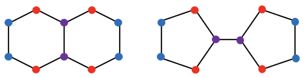

Figure S1. Topologically identical structure, decalin (left) and 1,1-bicyclopentane (right)

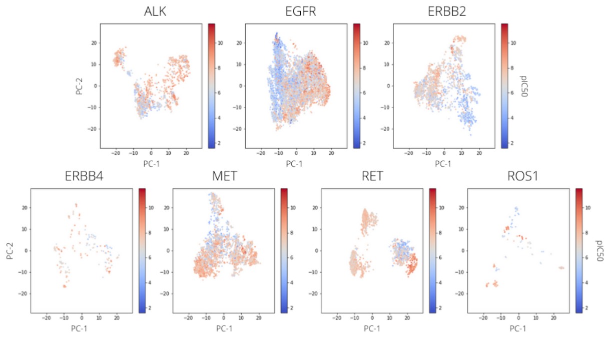

Figure S2. Two-dimensional PCA plots of train-validation sets before removing activity cliffs with pIC_{50} values presenting in color scales; the greater the values, the more potent the compounds are to the particular target. Each plot was separately prepared for specific target kinases.

1. Calculation of Activity Cliffs

Activity cliffs (ACs) are pairs or groups of compounds with high structural similarities with significant activity or potency differences. In the view of medicinal chemists, AC compounds provide essential Structure-Activity Relationship (SAR) structure information that remarkably led to the contribution of lead optimization and related works. However, the presence of discontinuous SAR regions in Quantitative SAR (QSAR) modelling entices adverse effects over the predictive ability of machine learning models [1–3]. Thus, we decided to add an AC-generator removal to our preprocessing procedure, exclusively for the model-building dataset. We adjusted the common AC-removal criterion using Tanimoto coefficient-based AC identification for this study. According to the reported recommendation [4], we considered compound pairs to be ACs under the basis that if (i) there was at least a 100-fold difference in potency values and (ii) Tanimoto similarity of Extended Connectivity Fingerprint 4 (ECFP4) reached the similarity threshold at 0.55. Tanimoto number of 0.55 from ECFP4 was applied since it has been identified for a high structural similarity in similarity-potency tree analysis [5], and reported in the usage with affinity value as K_i and IC_{50} [6].

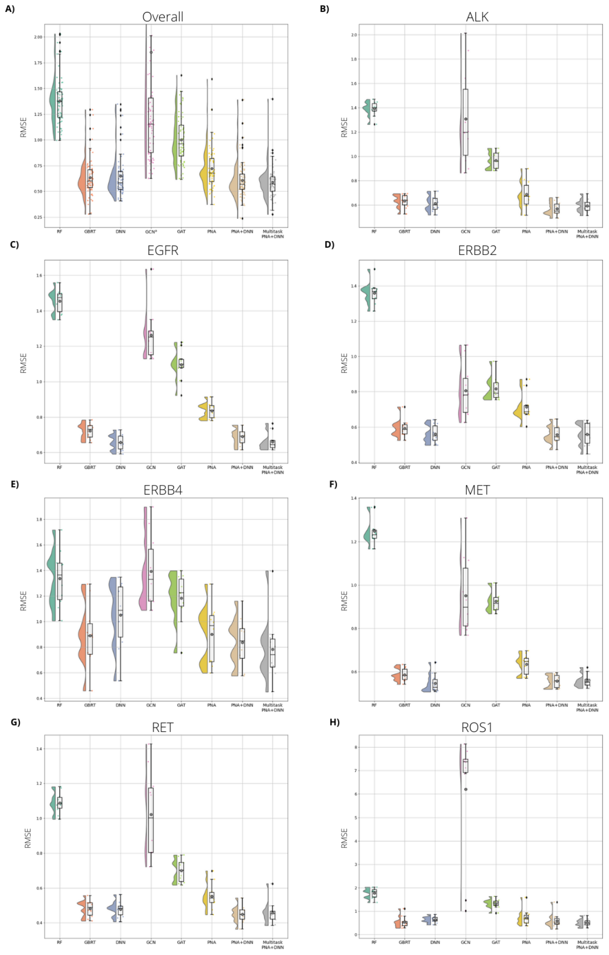

Figure S3. Benchmark model comparison: RF (dark cyan), GBRT (coral), DNN (light steel blue), GCN (pink), GAT (yellow green), PNA (gold), single-task PNA+DNN (tan) and multi-task PNA+DNN (light gray). In this graph, each model structure consists of all RMSE values from 10-fold cross-validation for overall (A), ALK (B), EGFR (C), ERBB2 (D), ERBB4 (E), MET (F), RET (G), and ROS1 (H) prediction. The RMSE distribution is shown in a half-violin plot and box plot, which shows quartiles 1, 2 and 3 of the distribution. The mean value of each model is marked as a grey dot inside the box plot. [a] Note that the overall graph (A) does not cover the RMSE points that present in the range of 6.5-8.5. for GCN.

2. Explanation of Online Screening Service

The online screening platform is available at https://github.com/kajjana/Multibind-RTKs with the step-by-step user guide for facilitating the discovery of crucial tyrosine kinase inhibitors involved in NSCLC therapy. There are two usage purposes, screening (Figure S6) and customized model training (Figure S7). For screening purposes, users are required to prepare a Comma-Separated Value (CSV) file containing the index and "smiles" column of screening molecules as the following example input (Figure S8). The input file needs to pass through the process as described in Github—get fingerprint, featurized, and predict applicability domain (AD).

* Correspondence: fahsain_pro@vistec.ac.th

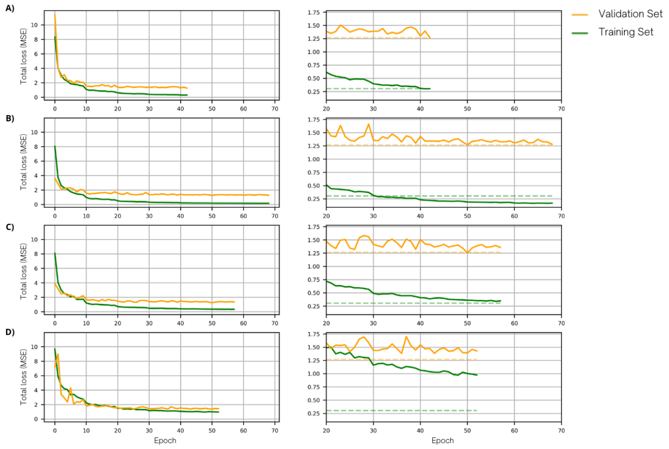

Figure S4. (Left panel) The graphs show the loss functions in mean-squared error (MSE) on training (green) and validation data (orange) over the running epochs of the model parameters used in this study (A) along with the varied parameters as follows: Dropout = 0.05, Weight Decay = 5×10^{-4} (B), Dropout = 0.2, Weight Decay = 5×10^{-5} (C) and Dropout = 0.5, Weight Decay = 10^{-6} (D). (Right panel) The graphs display the loss functions over zoomed-in epochs from 20 onwards. The solid lines indicate the MSEs during the training of each model. Meanwhile, the dash lines indicate the loss functions at the stopping point of the used model as a reference. This suggests that increasing regularization parameters for our model increase the validation loss value, which is considered an undesirable tradeoff due to the increase in the model's bias. The training curve also shows that the model was trained until the validation loss converged and did not over-trained.

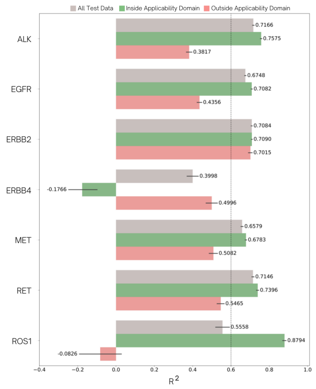

Figure S5. The bar graph presents the influence of the defined applicability domain to external-test activity prediction of 7 tyrosine kinases in R^2. Each target consists of 3 bars, which are denoted for all test data sets (grey), test compounds considered inside (green), and outside (pink) applicability domain. The error bar represents the 95%-confidential interval of the variation in predictive performance with 20 different random seeds. The numbers beside each error bar indicate the average R^2 over 20-times of test prediction. The dash lines indicate the predictive QSAR-model threshold at 0.6.

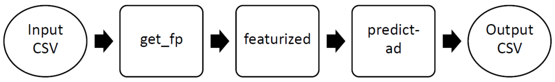

Figure S6. Screening workflow diagram

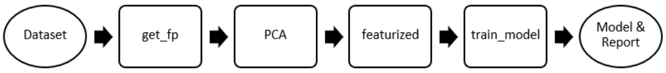

Figure S7. Customized model training and its prediction diagram

	smiles
0	O=C(Nc1ccc(Oc2ccnc3cc(-c4ccc(CN5CCNCC5)cc4)sc23)c(F)c1)N1CCN(c2ccccc2)C1=O
1	C=CC(=O)N1CCCC@@HC1
2	CN[C@@H]1C[C@H]2OC@@([C@@H]1OC)n1c3ccccc3c3c4c(c5c6ccccc6n2c5c31)C(=O)NC4
3	Cc1cccc(Nc2ncnc3ccc(Br)cc23)c1

Figure S8. The format of an input-CSV file for a screening process

	smiles	predicted_ pIC50_erbB4	predicted_ pIC50_egfr	predicted_ pIC50_met	predicted_ pIC50_alk	predicted_ pIC50_erbB2	predicted_ pIC50_ret	predicted_ pIC50_ros1	erbB4_ domain	egfr_ domain	met_ domain	alk_ domain	erbB2_ domain	ret_ domain	ros1_ domain
0	O=C(Nc1ccc(Oc2ccnc3cc(-c4ccc(CN5CCNCC5)cc4)sc23)c(F)c1)N1CCN(c2ccccc2)C1=O	7.26	7.21	7.52	7.21	6.94	7.56	7.62	outside	outside	inside	outside	outside	outside	outside
1	C=CC(=O)N1CCCC@@HC1	8.04	8.79	7.5	8.12	7.55	7.58	8.85	outside	inside	outside	inside	outside	inside	inside
2	CN[C@@H]1C[C@H]2OC@@([C@@H]1OC)n1c3ccccc3c3c4c(c5c6ccccc6n2c5c31)C(=O)NC4	8.13	7.95	7.33	9.61	7.84	10.07	11.19	outside	inside	outside	outside	inside	outside	outside
3	Cc1cccc(Nc2ncnc3ccc(Br)cc23)c1	6.87	6.49	7.36	5.99	5.8	6.27	6.87	inside	inside	outside	outside	outside	outside	outside

Figure S9. The format of an output-CSV file for a screening process

The output-CSV file containing pIC_{50}-activity prediction of 7 tyrosine kinases would be generated with the assigned AD presenting either "inside" or "outside" for each target shown in Figure S9. The promising candidates should be selected from high pIC_{50} compounds, and their AD should be considered "inside" for the targets of interest. For customized model training, users have to prepare a curated dataset, consisting of "smiles", "pIC50_erbB4", "pIC50_egfr", "pIC50_met", "pIC50_alk", "pIC50_erbB", "pIC50_ret", and "pIC50_ros1" columns. The pIC_{50} of some targets are allowed to be blanked if activity values have not been reported (Figure S10).

	smiles	pIC50_erbB4	pIC50_egfr	pIC50_met	pIC50_alk	pIC50_erbB2	pIC50_ret	pIC50_ros1
0	CS(=O)(=O)CCNCCCCOc1ccc2ncnc(Nc3ccc(F)c(Cl)c3)c2c1		7.7			6.68		
1	O=C(Nc1ccc(Oc2ccnc3cc(-c4ccc(CN5CCNCC5)cc4)sc23)c(F)c1)N1CCN(c2ccccc2)C1=O	7.66						
2	C=CC(=O)N1CCCC@@HC1		8.92					
3	CN[C@@H]1C[C@H]2OC@@([C@@H]1OC)n1c3ccccc3c3c4c(c5c6ccccc6n2c5c31)C(=O)NC4	7.55			8.77	7.47	9.34	10.15

Figure S10. The example of a model-using dataset in a CSV format for customized model training

The fingerprints need to be generated and passed through PCA and featurization before training. The PCA, pre-trained model and loss function report will be saved after the execution is finished. The report contains the loss of train and internal-test set over each training epoch. To utilize the customized model, users can follow the same manner as the screening scenario by using their pre-trained model instead of our provided pre-trained model. However, for the prediction of pIC_{50} using a customized pre-trained model, the AD analysis would be excluded as shown in Figure S11.

	smiles	predicted_ pIC50_erbB4	predicted_ pIC50_egfr	predicted_ pIC50_met	predicted_ pIC50_alk	predicted_ pIC50_erbB2	predicted_ pIC50_ret	predicted_ pIC50_ros1
0	CN1CCN(CCCN2c3ccccc3Sc3ccc(C(F)(F)F)cc32)CC1	5.22	4.52	6.2	6.23	4.68	6.33	6.64
1	Nc1ccc2oc(-c3ccccc3)cc(=O)c2c1	4.99	5.73	5.03	6.1	5.09	5.25	6.42
2	O=c1cc(-c2ccccc2)oc2ccc(O)cc12	6.38	8.7	6.04	5.77	5.7	4.51	6.89
3	COC(=O)c1ccc(NC(=O)CCC(=O)O)cc1	4.79	4.05	5.64	5.89	4.61	5.57	6.2

Figure S11. The example of an output-CSV file from the prediction using a customized model

References

1. Maggiora, G.M. On outliers and activity cliffs why QSAR often disappoints, 2006.
2. Cruz-Monteagudo, M.; Medina-Franco, J.L.; Perez-Castillo, Y.; Nicolotti, O.; Cordeiro, M.N.D.; Borges, F. Activity cliffs in drug discovery: Dr Jekyll or Mr Hyde? *Drug Discovery Today* **2014**, *19*, 1069–1080.
3. Stumpfe, D.; Hu, H.; Bajorath, J. Evolving concept of activity cliffs. *ACS omega* **2019**, *4*, 14360–14368.
4. Stumpfe, D.; Bajorath, J. Exploring activity cliffs in medicinal chemistry: miniperspective. *Journal of medicinal chemistry* **2012**, *55*, 2932–2942.
5. Wawer, M.; Bajorath, J. Similarity- potency trees: a method to search for SAR information in compound data sets and derive SAR rules. *Journal of chemical information and modeling* **2010**, *50*, 1395–1409.
6. Wassermann, A.M.; Dimova, D.; Bajorath, J. Comprehensive analysis of single-and multi-target activity cliffs formed by currently available bioactive compounds. *Chemical biology & drug design* **2011**, *78*, 224–228.
